# Educating the Educators

**DOI:** 10.1007/s13181-021-00827-6

**Published:** 2021-02-09

**Authors:** Lesley C. Pepin, Jennie A. Buchanan, Anthony F. Pizon, Kennon Heard, Matthew Zuckerman

**Affiliations:** 1 Emergency Medicine Residency, Denver Health and Hospital Authority, Denver, CO USA; 2 Rocky Mountain Poison & Drug Safety, Denver, CO USA; 3 Department of Emergency Medicine, Denver Health and Hospital Authority, Denver, CO USA; 4 Department of Emergency Medicine, University of Colorado, Aurora, CO USA; 5 Division of Medical Toxicology and Department of Emergency Medicine, University of Pittsburgh Medical Center, Pittsburgh, PA USA

**Keywords:** Education theory, Medical education, Curriculum, Toxicology Fellowship Training

Through emergency medicine’s evolution as a specialty, residency leadership embraced advanced training in educational theory and practice. It is not clear that leaders in medical toxicology training programs have followed this paradigm. Medical toxicologists have long been recognized as master educators with a range of experience educating fellows, residents, members of the public, and allied health care providers. However, enthusiasm and historic prowess in bedside and small group teaching may not translate to designing and running an outstanding medical toxicology training program.

While toxicology fellowship directors possess content expertise, there is a paucity of information on their training as educational leaders. We distributed a survey to fellowship directors via the American College of Medical Toxicology (ACMT) program director mailing listserv to assess participation and interest in formal activities focused on education and presented our findings at the 2020 American College of Medical Toxicology Annual Scientific Meeting. Only half of the responding program directors reported participation in faculty development activities focused on educational methodology, and less than a third reported development in educational theory specifically. Most reported an interest in more opportunities for educational training on theory and administration.

There is an opportunity and need to strengthen a field which constantly adapts to new circumstances and historically embraced advancements in medical education. Medical toxicology subspecialty training has long been the most common fellowship training for emergency medicine program directors; however, there has been a recent increase in the number of emergency medicine program directors who have completed advanced training or a fellowship in medical education [1]. Similar to residency leadership, medical toxicology program directors are an educational cornerstone, responsible for curriculum design, didactics, bedside education, and assessments. Despite this, medical toxicology fellowship directors of today seem similar to those of twenty years ago, often enthusiastic about education, but rarely receiving specialized training beyond a medical toxicology fellowship. Toxicology fellowship program directors must have three years of core faculty experience prior to assuming the fellowship director role, suggesting needed expertise beyond merely practicing toxicology [2, 3]. Is three additional years of practice enough?

Like the person in the burning building who’s more interested in putting out the fire than understanding the laws of thermodynamics, fellowship directors in our survey seem more focused on concrete educational issues. While the majority of current program directors support the need to understand educational milestones and curricular design, less than half agreed educational theory was vital to understand. This may suggest a misunderstanding of how educational theory informs development of effective curriculum and evaluation of milestone achievement. Much of the role of fellowship director relies on these areas of development. The Accreditation Council for Graduate Medical Education (ACGME) requires core faculty in toxicology must “devote a significant portion of their entire effort to fellow education and/or administration, and must, as a component of their activities, teach, evaluate, and provide formative feedback to fellows [3].”

Ironically, the same impulse which encourages someone to pursue advanced training in medical toxicology makes it less likely that they will also do a formal education fellowship or obtain an advanced degree in educational theory. New fellowship directors are frequently spending time running the fellowship or establishing their credentials in the field; many do not have the time to pursue formal education professional development until later in their career. More so, because they frequently attend meetings and conferences focused on toxicology, they are less likely to have the access or resources to independently implement professional development in formal education training. While residencies have assistant program directors who spend years preparing to lead the program by learning the nuances of the position, medical toxicology fellowships rarely have such a position. One program director commented, “Having been a program director for a long time, I think the administrative burdens have expanded tremendously. I’m not aware of any preparation for that aspect besides mentorship and the right disposition to get that work done." In our survey, we found most of those with more than five years of experience had participated in formal educational activities, unlike those with fewer years of experience. Enthusiasm for formal training is not lacking, as our survey suggests the more junior fellowship directors reported interest in such activities. The very time spent running a fellowship can improve educational skills, but a structured approach grounded in theory might provide greater impact and rigor. Examples where formal educational training may serve better than mere enthusiasm include remediating a poorly performing fellow or faculty member, curriculum assessment, and research on educational methodology [4, 5].

It is likely many emergency medicine fellowships, other than medical education, also need to embrace this key transition point and focus on program director development as lead educators within their field. Medical toxicology should shepherd this charge. We need to lead in this process by forming alliances with other national organizations (such as Council of Residency Directors (CORD) in Emergency Medicine, American College of Emergency Physicians (ACEP) Teaching Fellowship, Academic Life in Emergency Medicine, Harvard Macy Institute for Educators in Health Professions). Partnerships with our education expert colleagues (in emergency medicine or other specialties) may benefit further needs assessments to identify scope of the problem and approach to implementation of change.

Our national organizations should be a forum for further dissemination of educational training. The addition of educational sections at toxicologic national conferences could benefit medical toxicology faculty who are more likely to attend medical toxicology specific events. Implementation of online training sessions may allow a broader reach to faculty members of other emergency medicine subspecialties. Finally, the creation of a formal certificate program for early career medical toxicologists interested in education would allow for synchronous and asynchronous longitudinal sessions focused on education theory, curricular design, learner assessment, and remediation. Formal mentorship from senior faculty would be a source of support and pressure to complete such an optional curriculum, but over time such coursework could serve as a model for other fellowships.

There is an unmet need among our medical toxicology educational leaders. Establishing and supporting high standards for our educators is necessary to advance our specialty. Now is the time to take the next step, embrace early adaptation, and lead the way forward. We owe it to our patients, our learners, and our predecessors who inspired us to join this rarefied field.
